# Development, Preliminary Validation, and Refinement of the Composite Oral and Maxillofacial Pain Scale-Canine/Feline (COPS-C/F)

**DOI:** 10.3389/fvets.2019.00274

**Published:** 2019-08-22

**Authors:** Giorgia della Rocca, Alessandra Di Salvo, Maria Luisa Marenzoni, Enrico Bellezza, Giovanni Pastorino, Beatriz Monteiro, Paulo Steagall

**Affiliations:** ^1^Department of Veterinary Medicine, University of Perugia, Perugia, Italy; ^2^Department of Veterinary Medicine, CeRiDA (Centro di Ricerca sul Dolore Animale), University of Perugia, Perugia, Italy; ^3^Department of Clinical Sciences, Faculty of Veterinary Medicine, University of Montreal, Saint-Hyacinthe, QC, Canada

**Keywords:** cat, composite oral pain scale-canine/feline, COPS-C/F, dental, dog, oral, pain, scale

## Abstract

**Objectives:** Oral pain is underrecognized and undertreated in small animal practice. This study aimed to develop and perform a preliminary validation of an instrument to evaluate oral and maxillofacial pain in dogs and cats.

**Methods:** Indicators potentially associated with oral pain in dogs and cats were identified and selected. The Composite Oral Pain Scale-Canine/Feline (COPS-C/F) in the Italian language was developed using a two-part questionnaire (owner and veterinary specific questionnaires). The instrument was used to score the intensity of oral and maxillofacial pain in patients with oral disease. Content validity was performed and the COPS-C/F was applied to 20 dogs and 16 cats with oral disease at baseline and 15 days after dental treatment for construct validity. Criterion validity was assessed by comparing the COPS-C/F with a visual analog scale (VAS), a numeric rating scale (NRS), and a simple descriptive scale (SDS). Construct validity/responsiveness and criterion validity were assessed with Wilcoxon and Spearman Pearson tests, respectively (*p* ≤ 0.05). The Cronbach's alpha coefficient was used to calculate internal consistency. Thereafter, the instrument was refined and translated to English and back-translated for semantic equivalence.

**Results:** Construct validity was confirmed with a significant reduction of pain scores after treatment (*p* < 0.05) for most items. Criterion validity was confirmed by a significant correlation among the COPS-C/F total pain scores and those from VAS, NRS, and SDS (*p* < 0.05). Cronbach's alpha coefficient was 0.876 and 0.860 for the owner and the veterinary specific questionnaires, respectively, indicating good internal consistency. The items that did not present significant differences between time-points and the VAS, NRS, and SDS were removed prior to translation to English (COPS-C/F ENG).

**Conclusions and Clinical Relevance:** The study described the development and preliminary validation of the COPS-C/F as an instrument for pain assessment in dogs and cats. Refinement and back-translation of COPS-C/F with semantic equivalency resulted in the COPS-C/F ENG consisting of six and four items for the owner and veterinary specific questionnaires, respectively. The English version requires further validation and testing using a larger number of patients in the clinical setting.

## Introduction

Oral disease is one of the most common conditions in small animal practice (1). Oral and maxillofacial disorders that have been reported in the literature include gingivitis, periodontitis, feline odontoclastic resorptive lesions, ulcerative stomatitis, eosinophilic granuloma, oral tumors, palate cleft defects, lesions produced by dermatological conditions, abscesses, fractures, among others (2–5). These conditions have the capacity to induce oral pain and inflammation which may subsequently impact the patient's quality of life, nutritional status, and welfare (6). Veterinarians consider oral and maxillofacial disorders as an important cause of chronic pain in dogs (7), however studies on the subject are lacking and have mostly involved the use of local anesthetic techniques to reduce anesthetic requirements and provide perioperative analgesia in small animals (5, 8).

Pain scales or scoring systems can be used for assessment of acute and chronic pain in clinical practice. These tools including questionnaires may provide a cut-off score where interventional (rescue) analgesia is required and are used by veterinarians/owners for pain assessment in dogs (9–11) and cats (12–14). They involve a dynamic and interactive approach including observation of specific behaviors, palpation of the painful area, and subjective assessment of posture/position, among other subjective behavioral assessments. It is unknown how these instruments would “perform” in pain assessment of specific conditions such as oral pain, especially because signs of oral and maxillofacial pain have not been well-described and are not specifically included in these instruments.

Recognition and assessment of oral pain could allow early diagnosis and thus timely treatment of oral disease. A practical scoring system involving patient's assessment by both the owner and the veterinarian would provide valuable and thorough information on pain levels produced by oral conditions. Indeed, oral disease can be chronic in nature and might impact patient's health and behavior. In this context, this tool would have to go through validation for the assessment of its content, construct and criterion validity, responsiveness and reliability (15, 16).

The aim of this study was to develop and perform a preliminary validation of an instrument in the Italian language to evaluate oral and maxillofacial pain in dogs and cats, the Composite Oral Pain Scale-Canine/Feline (COPS-C/F). The hypothesis was that medical or surgical treatment of oral disease would reduce pain scores using the COPS-C/F at the follow-up visit when compared with scores upon presentation. Based on the results of preliminary validation, the instrument was refined and an English version of the scale is reported herein for future studies.

## Materials and Methods

This study was approved by the Bioethical Committee of the University of Perugia (protocol number 2017/25).

### Identification and Selection of Indicators of Oral Painful Conditions

The development of COPS-C/F was based on a literature search of review and original articles, and textbooks of oral disease and pathology that described clinical signs that could be related to oral pain (2–4, 6). Other indicators that could be indicative of oral pain based on clinical experience were also considered.

### Descriptive Levels and Attribution of Scores

The selected indicators were categorized in various descriptive levels (i.e., categories or sections) including a specific question for each level. Each indicator was then assigned an ordinal value (0–4). The items were assembled, and the first draft of the COPS-C/F in the Italian language was produced in a two-part questionnaire format, including separate questions for the pet owner and the veterinarian, respectively. This version was drafted by two authors (GP and GdR).

### Content Validity—Analysis by Experts

Content validity refers to the degree and adequacy with which the instrument actually measures the phenomenon of interest, in this case the pain (16, 17). It can be established based on the opinion of a committee of experts in the target field (16). Two veterinarians board-certified in veterinary dentistry and who were not involved in instrument development performed content (face) validity. These individuals performed an evaluation of the content and comprehensibility of each item and assigned whether the questions were relevant/important or not based on their experience. Procedures were described in lay-language when appropriate.

### COPS-C/F Prototype

The prototype version of COPS-C/F was composed of 13 items in the Italian language (Supplementary Material). Eight items were included in the owner specific questionnaire and were as follows: (1) feeding behavior according to food type (dry, wet, and mixed); (2) feeding behavior following changes in feeding (from dry to wet food); (3) feeding behavior according to food temperature which was subdivided into warm (3a), cold (3b), and room temperature (3c); (4) changes in feeding behavior compared with past experience; (5) changes in willingness to interact with other pets/people compared with past experience; (6) changes in grooming compared with past experience; (7) changes in activity/mobility compared with past experience; (8) presence of specific behaviors. Five items were included in the veterinary specific questionnaire and were as follows: (1) general evaluation; (2) nutritional status and muscle tone; (3) reaction to oral manipulation; (4) presence of specific signs during physical examination; (5) presence of specific signs during examination of oral cavity. In both parts of the COPS-C/F, a Visual Analog Scale (VAS) (18), a Numeric Rating Scale (NRS) (19), and a Simple Descriptive Scale (SDS) (20) were added as part of pain assessment to test the criterion validity of the instrument. The VAS consisted of a horizontal 10-cm line where “0” corresponded to “no pain” and “10” corresponded to the “worst imaginable pain” (18). The observer would place a mark on this line based on the level of pain he/she believed the animal was experiencing in which a continuous range of values was possible. The NRS is similar to the VAS, except that the horizontal line was numbered from 0 to 10; thus, only whole numbered values were possible (19). The SDS consisted of four degrees of pain intensity: absent, mild, moderate, severe where a value from 0 to 3 was assigned to each level, respectively. The observer would choose the most appropriate degree of pain intensity he/she believed the animal was experiencing (20).

Pain scores of the COPS-C/F ranged from 0 to 39 and from 0 to 30 for the owner and veterinary specific questionnaire, respectively.

### Clinical Application of COPS-C/F

Between June and September 2015 nine independent veterinarians recruited dogs and cats with oral conditions of different origin and severity (inclusion criteria). Each veterinarian enrolled from one to four dogs and from none to three cats. Animals were included in the study after obtaining owners' written informed consent. The owner was required to be available for a follow-up visit 15 days later. The presence of evident painful conditions other than the oral pathology represented the exclusion criterion used in the study.

Instructions about the inclusion and exclusion criteria and the timing of completion of the COPS-C/F were provided to veterinarians. After the initial consultation and physical examination, the instrument was completed by the owner and veterinarian.

A follow-up visit was scheduled exactly 15 days after the surgical treatment or beginning of the medical treatment, at which time the scale was once again completed for each patient by the owner and veterinarian.

### Construct Validity by Hypotheses Testing (Responsiveness)

Construct validity examines whether the instrument detects changes in the construct theoretically conjectured, which provides the strongest evidence for validation (21). Construct was defined herein as “presence of oral and maxillofacial pain.” One approach to assess construct validity is by means of responsiveness. It assesses the ability of the scale to detect a significant change in scores in an expected direction in responses to a predicted event that either increase or decrease scores (22). Thus, construct validity was verified by hypothesis testing assuming that medical or surgical treatment of the oral disease would reduce pain scores at the follow-up visit when compared with scores at presentation. Pain scores for each item and COPS-C/F total scores between the first and follow-up visit were thus compared.

### Criterion Validity by Comparison With Subjective Pain Scoring Systems

Criterion validity establishes the validity of a measuring instrument by comparing it with some external criterion which is usually considered a “gold-standard” (23). In the absence of a “gold-standard” measure to assess oral and maxillofacial pain in dogs and cats, criterion validity was assessed in comparison with three subjective pain scoring systems. Thus, it was determined by calculating the correlation among total scores of the COPS-C/F and scores obtained with VAS, NRS, and SDS (for owner and veterinarian separately).

### Internal Consistency

Internal consistency evaluates the correlation between different items of the same instrument. It measures whether several items that propose to measure the same general construct produce similar scores (16). Internal consistency of the COPS-C/F was thus evaluated.

### Statistical Analysis

Construct and criterion validity were assessed using the Wilcoxon test and the Spearman's rank correlation test, respectively; a statistically significant difference or correlation was considered when *p* ≤ 0.05. Internal consistency was evaluated using the Cronbach's alpha coefficient (24); values > 0.7 were considered acceptable (25).

Statistical analysis was carried out using a commercial software (SPSS, Statistics 24, IBM, Casalecchio di Reno, BO, Italy). All tests were performed analyzing data together and separately for dogs and cats.

### Refinement and English Translation of COPS-C/F

Based on the results of the validation process, refinement of the COPS-C/F was performed by removing items that did not show significant differences.

Then, the prototype instrument was translated from Italian to English by one of the authors (GdR) and reviewed by two individuals who were not involved in the development of COPS-C/F (BM and PS). The instrument was subsequently back-translated to Italian by another individual fluent in both Italian and English. The original instrument and the back-translated version in Italian were compared and minor adjustments were made in the English version (COPS-C/F ENG) to maintain semantic equivalence (26, 27).

## Results

Questionnaires were collected in 44 cases. Eight questionnaires were discarded due to missing data. Thirty-six questionnaires (20 dogs and 16 cats) were completed for both visits (first and follow-up visits).

Detailed demographic data, diagnosis and treatment received are presented in Tables 1, 2 for dogs and cats, respectively. In brief, dogs (different breeds, 11 females and 9 males, aged between 4 and 14 years old) were presented with gingivitis (*n* = 8), dental abscess (*n* = 1), dental fracture (*n* = 2) or avulsion (*n* = 1), periodontal disease (*n* = 3), apical granuloma (*n* = 2) and oral tumors (*n* = 3). Cats (all domestic short-hair, six females and 10 males, aged between 2 and 16 years old) were presented with stomatitis/faucitis/gingivostomatitis (*n* = 13), eosinophilic granuloma complex (*n* = 1), periodontal disease/feline odontoclastic resorptive lesions (*n* = 2). All dogs and seven cats underwent surgery including dental cleaning and/or dental extraction combined or not with medical treatment. Eleven dogs and 13 cats received medical treatment including the administration of antibiotics, corticosteroids and/or non-steroidal anti-inflammatory drugs as deemed necessary. None of the animals was treated with analgesics at the time of follow-up examination.

**Table 1:** Dogs' demographic data, diagnosis, and received treatment.

**Breed**	**Sex**	**Age (years)**	**Diagnosis**	**Treatment**
Yorkshire	Female	10	Gingivitis (from tartar accumulation)	Dental cleaning
Mongrel	Male	9	Gingivitis (from tartar and plaque accumulation)	Antibiotics, NSAIDs, dental cleaning, dental extraction
Miniature Schnauzer	Female	12	Gingivitis (from tartar and plaque accumulation) and dental mobility	Dental cleaning, extraction of mobile teeth
Mongrel	Female	14	Gingivitis (from tartar accumulation) and dental mobility	Antibiotics, dental cleaning, Extraction of incisor, and molar teeth
Mongrel	Male	4	Gingivitis (from tartar accumulation)	Dental cleaning, extraction of molar teeth
Mongrel	Female	14	Gingivitis (from tartar accumulation)	Antibiotics, non-steroidal anti-inflammatory drugs, dental cleaning
Pinscher	Male	9	Gingivitis (from tartar accumulation) and stomatitis	Antibiotics, dental cleaning, dental extraction
Mongrel	Female	7	Apical granuloma (4th superior right premolar)	Antibiotics, dental cleaning, dental extraction
Chihuahua	Male	5	Apical granuloma (1st superior right premolar), with sub-zygomatic fistula	Antibiotics, dental extraction, fistula closure with muscle-periosteal flap
Mongrel	Male	12	Tooth abscess	Antibiotics, NSAIDs, Dental cleaning, Dental extraction
Shiba inu	Male	8	Periodontal disease, multiple odontogenic fibromas	Fibrous extraction, extensive gingivectomy, dental cleaning, dental extraction
Dachshund	Male	13	Severe periodontal disease and oronasal fistula	Antibiotics, non-steroidal anti-inflammatory drugs, Dental cleaning, Dental extraction
Mongrel	Female	8	Gingivitis (from tartar and plaque accumulation)	Dental cleaning
Bolognese	Male	5	4th degree periodontitis, oronasal fistula, periapical pathology	Dental cleaning, dental extraction
Mongrel	Female	5	Complicated fracture (one tooth), periapical pathology (3 teeth, including the fractured teeth)	Antibiotics, non-steroidal anti-inflammatory drugs, dental extraction
Pointer	Female	7	Dental avulsion and alveolar bone fracture	Antibiotics, dental extraction
Mongrel	Female	11	Tooth fracture with periapical reaction	Dental extraction
Poodle	Male	5	High-grade undifferentiated mandibular sarcoma	Rostral mandibulectomy
Fox terrier	Male	8	Ameloblastoma	Excision of the neoplastic lesion
Labrador	Female	9	Mandibular osteosarcoma	Antibiotics, non-steroidal anti-inflammatory drugs, Tramadol, Hemimandibulectomy

**Table 2:** Cats' demographic data, diagnosis and received treatment.

**Breed**	**Sex**	**Age (years)**	**Diagnosis**	**Treatment**
Domestic short hair	Female	10	Stomatitis and faucitis	Corticosteroids
Domestic short hair	Male	4	Chronic faucitis	Corticosteroids
Domestic short hair	Male	6	Chronic faucitis/gingivitis	Antibiotics, corticosteroids
Domestic short hair	Male	12–13	Feline chronic gingivostomatitis	Antibiotics, corticosteroids, dental extraction
Domestic short hair	Male	12	Gingivitis/stomatitis	Antibiotics, corticosteroids
Domestic short hair	Female	13	Stomatitis	Antibiotics
Domestic short hair	Male	9	Gingivitis	Antibiotics
Domestic short hair	Male	8	Lymphoplasmacytic stomatitis	Antibiotics, extraction of molar, and premolar teeth
Domestic short hair	Female	7	Feline chronic gingivostomatitis	Dental extraction
Domestic short hair	Male	10	Feline chronic gingivostomatitis	Dental extraction
Domestic short hair	Female	16	Gingivitis (from tartar and plaque accumulation)	Antibiotics, non-steroidal anti-inflammatory drugs
Domestic short hair	Female	6	Periodontal disease, feline odontoclastic resorptive lesion	Antibiotics, extraction of the involved teeth, dental cleaning, curettage
Domestic short hair	Male	16	Diffuse feline odontoclastic resorptive lesion	Extraction of the involved teeth, alveolar osteoplastic
Domestic short hair	Male	4	Gingivitis/glossitis (suspected calicivirus infection)	Antibiotics, non-steroidal anti-inflammatory drugs, dental extraction
Domestic short hair	Female	2	Ulcerative gingivitis/glossitis (suspected calicivirus infection)	Antibiotics
Domestic short hair	Male	6	Eosinophilic granuloma complex (indolent ulcer, eosinophilic plaque)	Antibiotics, corticosteroids

### Construct Validity by Hypotheses Testing (Responsiveness)

COPS-C/F pain scores obtained for each item of the scale were significantly decreased at follow-up visit when compared with the first visit in six out of eight items in the owner specific questionnaire, and in five out of five items of the veterinary specific questionnaire when data for dogs and cats were analyzed together (*p* < 0.05), hence supporting construct validity (responsiveness) of the instrument. “Feeding behavior following changes in feeding (from dry to wet food)” and “feeding behavior according to food temperature” were not significantly different between the two visits.

Similar results were observed when data for cats and dogs were analyzed separately. However, the item “nutritional status and muscle tone” in the veterinary specific questionnaire did not show any significant difference between the first and follow-up visits in dogs.

Total scores obtained by the sum of all items' scores (owner and veterinary specific questionnaire) were also significantly decreased at follow-up visit when compared with first visit when data were evaluated for both species together [owner's median scores: 8 (first visit) and 2 (follow-up); veterinarian's median scores: 8 (first visit) and 2 (follow-up)] or separately [owner's median scores for cats: 11 (first visit) and 3 (follow-up); owner's median scores for dogs: 7 (first visit) and 1 (follow-up); veterinarian's median scores for cats: 11 (first visit) and 3 (follow-up); veterinarian's median scores for dogs: 6 (first visit) and 1 (follow-up)].

Tables 3, 4 show median and range (minimum and maximum) scores of each item of the scale and total scores recorded by owners and veterinarians, respectively.

**Table 3:** Owners' scores of each item and the total score of the COPS-C/F.

**Visit**	**Item**	**Dogs and cats**		**Cats only**		**Dogs only**	
		**Median (range)**	***P* value**	**Median (range)**	***P* value**	**Median (range)**	***P* value**
First	Q1	1 (0–4)	0.000	2.5 (0–4)	0.001	0 (0–4)	0.020
Follow-up	Q1	0 (0–1)		0 (0–1)		0 (0–1)	
First	Q2	1 (0–2)	0.479	0.5 (0–2)	0.317	1 (0–2)	0.144
Follow-up	Q2	0 (0–2)		0.5 (0–2)		0 (0–2)	
First	Q3a	0 (0–2)	0.157	0 (0–1)	0.564	0 (0–2)	0.180
Follow-up	Q3a	0 (0–1)		0 (0–1)		0 (0–1)	
First	Q3b	0 (0–4)	0.026	0 (0–4)	0.180	0 (0–4)	0.066
Follow-up	Q3b	0 (0–1)		0 (0–1)		0 (0–1)	
First	Q3c	0 (0–3)	0.066	0 (0–2)	0.180	0 (0–3)	0.157
Follow-up	Q3c	0 (0–1)		0 (0–1)		0 (0–1)	
First	Q4	1 (0–3)	0.001	1 (0–3)	0.007	0 (0–3)	0.026
Follow-up	Q4	0 (0–2)		0 (0–2)		0 (0–2)	
First	Q5	1 (0–4)	0.000	1.5 (0–4)	0.004	1 (0–2)	0.013
Follow-up	Q5	0 (0–1)		0 (0–1)		0 (0–1)	
First	Q6	0 (0–2)	0.001	1 (0–1)	0.009	0 (0–2)	0.046
Follow-up	Q6	0 (0–1)		0 (0–2)		0 (0–1)	
First	Q7	0.5 (0–2)	0.001	0.5 (0–2)	0.003	0 (0–2)	0.011
Follow-up	Q7	0 (0–1)		0 (0–1)		0 (0–1)	
First	Q8	2 (0–6)	0.000	2 (1–6)	0.002	2 (0–5)	0.000
Follow-up	Q8	0 (0–3)		1 (0–3)		0 (0–2)	
First	Total score	8 (1–24)	0.000	11 (2–21)	0.001	7 (1–24)	0.000
Follow-up	Total score	2 (0–8)		3 (0–8)		1 (0–6)	

*Scores were given by owners at the first and at follow-up visits and the p-values were obtained from the comparison between these two time-points. Values are presented as median and range (minimum and maximum). Statistically significant difference: p ≤ 0.05. Q, question; COPS-CF, Composite Oral Pain Scale-Canine/Feline*.

**Table 4:** Veterinarians' scores of each item and the total score of the COPS-C/F.

**Visit**	**Item**	**Dogs and cats**		**Cats only**		**Dogs only**	
		**Median (range)**	***P* value**	**Median (range)**	***P* value**	**Median (range)**	***P* value**
First	Q1	1 (0–4)	0.000	3 (1–4)	0.004	1 (0–4)	0.004
Follow-up	Q1	1 (0–3)		1 (0–2)		1 (0–3)	
First	Q2	0 (0–2)	0.025	1 (0–2)	0.025	1 (1–2)	1.000
Follow-up	Q2	0 (0–2)		0 (0–2)		0 (0–2)	
First	Q3	2 (0–4)	0.000	2 (0–4)	0.007	2 (0–4)	0.001
Follow-up	Q3	0 (0–4)		1 (0–2)		0 (0–4)	
First	Q4	1 (0–6)	0.000	1 (0–3)	0.002	1 (0–6)	0.001
Follow-up	Q4	0 (0–2)		0 (0–2)		0 (0–1)	
First	Q5	3 (1–5)	0.000	3 (0–5)	0.001	3 (1–5)	0.000
Follow-up	Q5	0 (0–4)		1 (0–4)		0 (0–1)	
First	Total score	8 (4–17)	0.000	11 (4–15)	0.001	6 (4–17)	0.000
Follow-up	Total score	2 (0–10)		3 (1–10)		1 (0–7)	

*Scores were given by veterinarians at the first and at follow-up visits and the p-values were obtained from the comparison between these two time-points. Values are presented as median and range (minimum and maximum). Statistically significant difference: p ≤ 0.05. Q, question; COPS-CF, Composite Oral Pain Scale-Canine/Feline*.

### Criterion Validity by Comparison With Three Subjective Pain Scoring Systems

Tables 5, 6 show the owners' and veterinarians' scores for VAS, NRS, and SDS, respectively. There was a significant (from moderate to high) correlation between total COPS-C/F scores and VAS, NRS, and SDS scores given by owners and veterinarians both at the first and follow-up visits, hence supporting criterion validity (Table 7).

**Table 5:** Owners' scores for VAS, NRS, and SDS.

**Visit**	**Scale**	**Dogs and cats**	**Cats only**	**Dogs only**
		**Median (range)**	**Median (range)**	**Median (range)**
First	VAS	5.7 (0.2–10)	6 (0.9–10)	4.3 (0.2–9.2)
Follow-up	VAS	0.5 (0–6)	0.5 (0–5)	0.6 (0–5.6)
First	NRS	6 (1–10)	7 (1–10)	5 (1–10)
Follow-up	NRS	1 (1–7)	1 (1–6)	1 (0–7)
First	SDS	3 (1–4)	3 (1–4)	3 (1–4)
Follow-up	SDS	1 (1–3)	1 (1–3)	1 (1–3)

*Scores were given by owners at the first and at follow-up visits and the p values were obtained from the comparison between these two time-points. Values are presented as median and range (minimum and maximum). VAS, Visual Analog Scale; NRS, Numeric Rating Scale; SDS, Simple Descriptive Scale*.

**Table 6:** Veterinarians' scores of VAS, NRS, and SDS.

**Visit**	**Scale**	**Dogs and cats**	**Cats only**	**Dogs only**
		**Median (range)**	**Median (range)**	**Median (range)**
First	VAS	4 (0.1–10)	4.9 (1.5–10)	3.8 (0.1–9.3)
Follow-up	VAS	0.5 (0–4.7)	1.2 (0–4.7)	0.4 (0–2.4)
First	NRS	5 (1–10)	6 (2–10)	4 (1–9)
Follow-up	NRS	1 (1–6)	2 (1–6)	1 (1–3)
First	SDS	2 (0–4)	2 (1–4)	2 (0–3)
Follow-up	SDS	0 (0–2)	1 (0–2)	0 (0–1)

*Scores were given by veterinarians at the first and at follow-up visits and the p values were obtained from the comparison between these two time-points. Values are presented as median and range (minimum and maximum). VAS, Visual Analog Scale; NRS, Numeric Rating Scale; SDS, Simple Descriptive Scale*.

**Table 7:** Correlation between the COPS-C/F total score and the VAS, NRS, and SDS.

	**First visit**			**Follow-up visit** **(15 days after treatment)**		
	**VAS**	**NRS**	**SDS**	**VAS**	**NRS**	**SDS**
Owners'	0.718*	0.766*	0.678*	0.516*	0.495*	0.570*
Veterinarians'	0.615*	0.712*	0.460	0.515*	0.521*	0.570*

* p ≤ 0.005.

*Interpretation of Spearman's correlation coefficient: 0–0.35: low correlation; 0.35–0.7: moderate correlation; 0.7–1.0: high correlation. VAS, Visual Analog Scale; NRS, Numeric Rating Scale; SDS, Simple Descriptive Scale; COPS-CF, Composite Oral Pain Scale-Canine/Feline*.

### Internal Consistency

The Cronbach's alpha coefficient for the scale's total score was 0.876 and 0.860 for the owner and the veterinary specific questionnaire, respectively, indicating good internal consistency.

### Refinement and English Translation of COPS-C/F

The items that did not show significant differences between time-points (i.e., “feeding behavior following changes in feeding—from dry to wet food” and “feeding behavior according to food temperature” from the owner specific questionnaire and “nutritional status and muscle tone” from the veterinary specific questionnaire) were removed from the COPS-C/F prior to translation and back-translation. The VAS, NRS, and SDS scores were also removed since they had only been included to test the criterion validity of the COPS-C/F.

The final COPS-C/F ENG is shown in Table 8.

**Table 8:** COPS-C/F ENG: English version after refinement.

**Composite oral pain scale-canine/feline (COPS-C/F ENG)**	
***Owner Specific Questionnaire***	
**1st Evaluation**	**Follow-up**
**What type of food do you give to your pet?**	
	Dry
	Wet
	Mixed
**Q1. Considering the food that you give and compared with the past, your pet:**	
0	Eats normally
1	Eats more slowly
2	Eats less
3	Has some unusual behaviors (e.g., retracts/complains while chewing; drops the bite while chewing, other)
4	Does not eat at all
**Q2. Have you noticed any change in your pet's feeding behavior compared with the past?**	
0	No change
1	Shows interest in food but after a few bites goes away from it
2	Shows less interest in food
3	It is uninterested in food
**Q3. Have you noticed any change in your pet's willingness to interact/play with people or other pets compared with the past?**	
0	No change
1	Less active than usual, but still willing to interact/play
2	Depressed, less willing to interact/play
3	Nervous, anxious, sometimes aggressive toward people/other pets
4	No longer interacts with people/other pets; tends to hide or to lay in its kennel
**Q4. Have you noticed any change in your pet's personal hygiene (grooming, licking) compared with the past?**	
0	No change
1	Spends less time cleaning itself
2	Does not clean itself (it is dirty; the fur is disheveled)
**Q5. Have you noticed any change in your pet's physical activity/mobility (running, walking, etc.) compared with the past?**	
0	No change
1	Less willing to do physical activity
2	Refuses to do any physical activity
**Q6. Have you noticed the presence of one or more of the following behaviors?**	
1	Moans/groans
1	Increased aggressiveness and/or nervousness
1	Avoids being touched around nose/mouth
1	Has less interest in playing involving the use of mouth (wooden sticks, toys, etc.)
1	Often scratches the mouth area
1	Has difficulties in yawning and/or opening the mouth
1	Produces more saliva and/or swallows more frequently
1	Bad breath
1	Grinds its teeth
1	Chewing without food in its mouth
***Veterinary Specific Questionnaire***	
**Q1. The animal is:**	
0	Lively, happy
1	Quiet
2	Indifferent to its surroundings
3	Nervous, anxious, scared
4	Depressed, unresponsive to stimuli
**Q2. When manipulating the oral cavity, the animal is:**	
0	Calm, relaxed
1	Looks around
2	Tries to avoid manipulations
3	Complains
4	Growls/hisses and/or attempts to bite/scratch
**Q3. During the visit, did you observe any of the following?**	
1	Ptyalism
1	Nasal discharge
1	Resistance/difficulty in opening the mouth
1	Crackles when manipulating the temporomandibular joint
1	Atrophy of masseter/temporalis muscles
1	Swelling of masseter/temporalis muscles
1	Mouth swelling or asymmetry
1	Sensitivity and/or increase in resistance to the eye pressure
**Q4. While examining the oral cavity, did you observe any of the following?**	
1	Accumulation of food in the oral cavity
1	Halitosis
1	Spontaneous/provoked gums bleeding
1	Dental fractures
1	Dental malformations
1	Tooth discoloration
1	Tooth mobility
1	Enamel hypocalcification or hypoplasia
1	Hyperplasia of the gums or presence of gum tumors
1	Ulcerative lesions of the oral mucosa/tongue/palate/gums
1	Maxillary or peri-zygomatic fistulas
1	Gum or mucosae fistulas

## Discussion

Oral and maxillofacial disorders can be painful and result in chronic suffering if left untreated. Thus, appropriate recognition of oral pain is essential for timely treatment of these conditions (6). This study presented the development, preliminary validation and refinement of an instrument to evaluate oral painful conditions in dogs and cats by owners and veterinarians.

Validation of health measurement scales should ensure that the instrument measures what it is intended to measure and includes content, construct and criterion validity (12). After content validity, the majority of items in the prototype (Italian) version of COPS-C/F showed appropriate construct and criterion validity, and good internal consistency.

Construct validity refers to the degree to which a score can be interpreted as representing the intended underlying construct. In other words, it refers to the extent to which a measure assesses the construct of interest (15, 25). In this study, construct was defined as “presence of oral and maxillofacial pain.” Construct validity can be assessed by different approaches. One approach refers to the ability of the instrument to detect differences between groups or, in this case, to distinguish between painful and non-painful individuals. Thus, animals in a control group (non-painful) would have significantly lower pain scores than painful ones. The lack of a control group is an important limitation and a source of intrinsic bias in this study. Another approach for assessing construct validity is to test the responsiveness of the scale (i.e., treatment effect). Thus, pain scores in painful animals would decrease after treatment (15). In this study, the COPS-C/F showed appropriate responsiveness to medical or surgical treatment of oral disease since pain scores were decreased at the follow-up visit when compared with the first presentation. However, a placebo effect may have been observed since owners and veterinarians where aware that animals were being treated (i.e., observer bias). Indeed, veterinarians completing the COPS-C/F were the ones performing or prescribing treatment and reevaluating these animals. This represents another significant limitation of this study. Future studies should minimize bias by including a control group of non-painful animals and by including independent observers who are blinded to disease severity and/or treatment. On the other hand, the scale showed promising results despite having nine different veterinarians evaluating a small number of patients each. This could be considered a strength of the study although it certainly added variability to the data.

Criterion validity was observed with the significant correlation between COPS-C/F total score (dogs and cats together) and subjective pain scoring systems (VAS, NRS, and SDS) at first and follow-up visits. It is arguable that criterion validity should be performed against a “gold-standard” instrument that has been validated for the same purposes. However, the prototype instrument was developed in the Italian language and similar instruments for assessment were not available in the same language until recently (28). In addition, there is currently no “gold-standard” available for assessment of oral and maxillofacial pain. In the absence of such instrument, it was decided to use subjective pain scoring systems such as the VAS, SDS and NRS for comparison because they can be easily applied to owners.

The reliability testing of an instrument involves internal consistency, intra-rater and inter-rater testing (12). Preliminary validation of COPS-C/F showed good internal consistency. Further studies involving video or real-time pain assessment of the same patient by different raters may allow the evaluation of inter-rater reliability, or the ability of the instrument to produce similar results when used by different individuals (12). Similarly, intra-rater reliability could be performed using video-assessment when the same individual (rater) evaluates the same patient at different times to test the ability of the instrument to produce similar results when used by the same individual (12).

A particular advantage of the COPS-C/F over previous instruments for pain assessment is that veterinarians and/or owners can complete the evaluation of pain in an animal with a specific condition (i.e., oral disease). However, the discriminatory ability of this instrument has not been investigated and it is not possible to differentiate pain intensity (mild, moderate, and severe) or painful vs. non-painful individuals. The ability of COPS-C/F to be used as a monitoring tool in the clinical setting and to identify “treatment failure” requires further evaluation to ensure that the instrument is practical, user-friendly and easy to implement independently of the observer (owner or veterinarian) or origin and type of pain (e.g., medical or surgical). Moreover, the identification of a cut-off for interventional (rescue) analgesia (sensitivity testing) should be the subject of future studies.

The COPS-C/F ENG can now undergo further validation and testing in clinical trials using a larger number of patients, thus providing veterinarians with a potential scale for canine and feline oral pain assessment. Further content validity, construct validity, criterion validity using another validated instrument (e.g., the Glasgow canine or feline pain scale), inter- and intra-rater reliability, responsiveness including a control group and sensitivity (discriminatory ability) testing of COPS-C/F ENG are warranted.

## Data Availability

The datasets generated for this study are available on request to the corresponding author.

## Ethics Statement

This study was approved by the Bioethical Committee of the University of Perugia (protocol number 2017/25). The owners signed a written informed consent.

## Author Contributions

GR participated in the conceptualization of the study, coordinated the clinical phase, and drafted the manuscript. AS assisted in revising the final manuscript. MM performed the statistical analysis. EB participated in the study design and supervised the clinical phase. GP participated in the distribution and returning of the questionnaires and data collection. BM and PS participated in the refinement of the COPS-C/F and manuscript drafting and editing. All authors read and approved the final manuscript.

### Conflict of Interest Statement

The authors declare that the research was conducted in the absence of any commercial or financial relationships that could be construed as a potential conflict of interest.

## Abbreviations

COPS-C/F ENG: composite oral pain scale-canine/feline English version
COPS-C/F: composite oral pain scale-canine/feline
NRS: numeric rating scale
SDS: simple descriptive scale
VAS: visual analog scale.
