# Supplementary material for: Development, Preliminary Validation, and Refinement of the Composite Oral and Maxillofacial Pain Scale-Canine/Feline (COPS-C/F)
Source: Front Vet Sci. 2019 Aug 22;6:274. doi: 10.3389/fvets.2019.00274 (PMC6714595; doi:10.3389/fvets.2019.00274)
Supplement: Supplementary file 1 [file Table_1.DOCX]

# Questionario per la valutazione del dolore orale nel cane e nel gatto (COPS-C/F)

*Parte per il proprietario*

🗆 1a VALUTAZIONE 🗆 FOLLOW-UP

| **Che tipo di alimento somministri al tuo animale?** | |
| --- | --- |
|  | Secco |
|  | Umido |
|  | Misto |
| **D1. Rispetto all’alimento fornito e rispetto al passato, il tuo animale:** | |
| 0 | Mangia normalmente |
| 1 | Mangia più lentamente |
| 2 | Mangia di meno |
| 3 | Ha dei comportamenti anomali (es. si retrae/si lamenta dopo qualche atto masticatorio; lascia cadere il boccone dopo qualche atto masticatorio; altro) |
| 4 | Non mangia |
| **D2. Nel caso avessi tentato di cambiare l’alimento da secco a umido, hai notato un miglioramento nell’appetito del tuo animale?** | |
| 0 | Non ho effettuato questo cambiamento |
| 1 | Si |
| 2 | No |
| **L’alimento viene dato al tuo animale:** | |
|  | Caldo |
|  | Freddo |
|  | A temperatura ambiente |
| **D3. Hai notato delle differenze riguardo all’alimentazione del tuo animale in rapporto alla temperatura dell’alimento?** | |
| **D3a. Se caldo:** | |
| 0 | Nessuna differenza |
| 1 | Mangia più lentamente |
| 2 | Mangia di meno |
| 3 | Ha dei comportamenti anomali (es. si retrae/si lamenta dopo qualche atto masticatorio; ha difficoltà nel prendere l’alimento dalla ciotola; lascia cadere il boccone dopo qualche atto masticatorio; altro) |
| 4 | Non mangia |
| **D3b. Se freddo (considerare per questa domanda anche l’ acqua ):** | |
| 0 | Nessuna differenza |
| 1 | Mangia/Si abbevera più lentamente |
| 2 | Mangia/Si abbevera di meno |
| 3 | Ha dei comportamenti anomali (es. si retrae/si lamenta dopo qualche atto masticatorio; lascia cadere il boccone dopo qualche atto masticatorio; altro) |
| 4 | Non mangia/Non beve |
| **D3c. Se a temperatura ambiente:** | |
| 0 | Nessuna differenza |
| 1 | Mangia più lentamente |
| 2 | Mangia di meno |
| 3 | Ha dei comportamenti anomali (es. si retrae/si lamenta dopo qualche atto masticatorio; lascia cadere il boccone dopo qualche atto masticatorio; altro) |
| 4 | Non mangia |
| **D4. Hai notato rispetto al passato variazioni del comportamento del tuo animale quando si alimenta?** | |
| 0 | Nessuna variazione |
| 1 | Mostra interesse verso il cibo ma dopo qualche boccone se ne allontana |
| 2 | Mostra meno interesse verso il cibo |
| 3 | È disinteressato al cibo |
| **D5. Hai notato variazioni nella voglia del tuo animale di interagire/giocare con persone/altri animali rispetto al passato?** | |
| 0 | Nessuna variazione |
| 1 | È meno attivo del solito, ma comunque disponibile all’interazione/gioco |
| 2 | È depresso, è poco interessato all’interazione/gioco |
| 3 | È nervoso, ansioso, a volte aggressivo verso persone/altri animali |
| 4 | Non interagisce più con persone/altri cani, tende a nascondersi o a stare nella propria cuccia |
| **D6. Hai notato dei cambiamenti riguardanti l’igiene personale del tuo animale (pulirsi, leccarsi) rispetto al passato?** | |
| 0 | Nessun cambiamento |
| 1 | Dedica meno tempo all’igiene personale |
| 2 | Non effettua più azioni dedite all’igiene personale (ha il pelo sporco, arruffato) |
| **D7. Hai notato cambiamenti riguardo l’attività fisica del tuo animale (camminare, correre, ecc.) rispetto al passato?** | |
| 0 | Nessun cambiamento |
| 1 | È meno disposto ad effettuare attività fisica |
| 2 | Si rifiuta di compiere attività fisica |
| **D8. Hai notato, nel tuo animale, la presenza di uno o più dei seguenti atteggiamenti?** | |
| 1 | Guaiti/lamenti |
| 1 | Maggiore aggressività e nervosismo |
| 1 | Evita di farsi toccare il muso/la bocca |
| 1 | Ha meno interesse verso giochi che prevedono l’uso della bocca (bastoncini di legno, pupazzi etc.) |
| 1 | Si gratta spesso la zona della bocca |
| 1 | Ha difficoltà a sbadigliare e/o ad aprire la bocca |
| 1 | Produce più saliva e/o deglutisce più frequentemente |
| 1 | Alito maleodorante |
| 1 | Digrigna i denti |
| 1 | Mastica a vuoto |
| **Indica quello che, secondo te, è il livello di dolore percepito dal tuo animale, apponendo una X su uno dei numeri riportati (considera ‘’1’’ come ‘’assenza di dolore’’ e ‘’10’’ come ‘’ il peggior dolore immaginabile’’)**   \| 1 \| 2 \| 3 \| 4 \| 5 \| 6 \| 7 \| 8 \| 9 \| 10 \| \| --- \| --- \| --- \| --- \| --- \| --- \| --- \| --- \| --- \| --- \| | |
| **Indica quello che, secondo te, è il livello di dolore percepito dal tuo animale, apponendo una X sulla barra sottostante (considera l’estremità sinistra come ‘’assenza di dolore’’ e quella destra come ‘’il peggior dolore immaginabile’’)**  0mm 100mm | |
| **Indica quello che, secondo te, è il livello di dolore percepito dal tuo animale** | |
| 1 | Nessun dolore |
| 2 | Dolore lieve |
| 3 | Dolore moderato |
| 4 | Dolore severo |

*Parte per il veterinario*

🗆 1a VALUTAZIONE 🗆 FOLLOW-UP

| **D1. L’animale è:** | |
| --- | --- |
| 0 | Vivace, felice |
| 1 | Quieto |
| 2 | Indifferente a ciò che lo circonda |
| 3 | Nervoso, ansioso, spaventato |
| 4 | Depresso, non risponde agli stimoli |
| **D2. Descrivi lo stato di nutrizione e la tonicità muscolare dell’animale:** | |
| 0 | Nella norma |
| 1 | Magro |
| 2 | Cachettico |
| **D3. Manipolando la cavità orale, l’animale:** | |
| 0 | È sereno, rilassato |
| 1 | Si guarda attorno |
| 2 | Cerca di sottrarsi alla manipolazione |
| 3 | Si lamenta |
| 4 | Ringhia/soffia e/o tenta di mordere/graffiare |
| **D4. Alla visita clinica, hai osservato alcuni di questi sintomi/segni clinici?** | |
| 1 | Scialorrea |
| 1 | Scolo nasale |
| 1 | Resistenza/difficoltà all’apertura della bocca |
| 1 | Crepitii alla manipolazione dell’articolazione temporo-mandibolare |
| 1 | Atrofia dei muscoli masseteri e/o temporali |
| 1 | Gonfiore dei muscoli masseteri e/o temporali |
| 1 | Tumefazione o asimmetrie del muso |
| 1 | Sensibilità e/o aumento della resistenza alla digitopressione oculare |
| **D5. All’esame particolare del cavo orale, hai osservato alcuni di questi sintomi/segni clinici?** | |
| 1 | Ristagno di alimento in cavità orale |
| 1 | Alitosi |
| 1 | Sanguinamento gengivale spontaneo/provocato |
| 1 | Fratture dentali |
| 1 | Malformazioni dentali |
| 1 | Discromie dentali |
| 1 | Mobilità dei denti |
| 1 | Ipocalcificazione o ipoplasia dello smalto |
| 1 | Iperplasia o presenza di neoformazioni (tumori) gengivali |
| 1 | Lesioni ulcerative su mucosa orale/lingua/palato/gengive |
| 1 | Fistole sottozigomatiche o mascellari |
| 1 | Fistole gengivali o mucosali |
| **Indica quello che, secondo te, è il livello di dolore percepito dall’animale, apponendo una X sulla scala sottostante (considera ‘’1’’ come ‘’assenza di dolore’’ e ‘’10’’ come ‘’ il peggior dolore immaginabile’’)**   \| 1 \| 2 \| 3 \| 4 \| 5 \| 6 \| 7 \| 8 \| 9 \| 10 \| \| --- \| --- \| --- \| --- \| --- \| --- \| --- \| --- \| --- \| --- \| | |

| **Indica quello che, secondo te, è il livello di dolore percepito dall’animale, apponendo una X sulla barra sottostante (considera l’estremità sinistra come ‘’assenza di dolore’’ e quella destra come ‘’il peggior dolore immaginabile’’)**  0mm 100mm |
| --- |
| **Indica quello che, secondo te, è il livello di dolore percepito dall’animale** |

| 0 | Nessun dolore |
| --- | --- |
| 1 | Dolore lieve |
| 2 | Dolore moderato |
| 3 | Dolore severo |
